# Enhancing Bone Repair Process: Application and Perspective on Photothermal Materials

**DOI:** 10.3390/molecules31132299

**Published:** 2026-07-01

**Authors:** Xuchen Yan, Chuanpeng Zhou, Hanyue Mao, Kunlu Lin, Ying Yang, Haoming Liu, Long Liu, Xiaoyan Wang

**Affiliations:** College of Science, National University of Defense Technology, Changsha 410073, China; yanxuchen23@163.com (X.Y.); zhouchuanpeng22@163.com (C.Z.); maohanyue22@163.com (H.M.); kunlulin@163.com (K.L.); yangying5952@163.com (Y.Y.); lhm@nudt.edu.cn (H.L.); llbio@126.com (L.L.)

**Keywords:** bone repair, photothermal therapy, mild hyperthermia, immune regulation, sequential repair

## Abstract

Repairing large bone defects remains a clinical challenge in orthopedics. Near-infrared (NIR) photothermal therapy (PTT) has recently expanded from high-temperature tumor ablation to the field of mild bone regeneration. Maintaining temperatures within a mild window of 40–42 °C accelerates bone healing by activating osteogenic signals, modulating the immune microenvironment, and providing antibacterial effects. It is important to note that the therapeutic efficacy is highly dependent on the precise control of both temperature and exposure duration: temperatures exceeding 42–43 °C can induce cell apoptosis, while temperatures above 45 °C typically cause necrosis. The reviewed studies employed controlled exposure times (typically 5–15 min per session) to maintain cell viability above 85%, with functional assessments confirming preserved osteogenic differentiation capacity of bone marrow-derived mesenchymal stem cells (BMSCs) and maintained macrophage plasticity after mild photothermal treatment. This performance depends on photothermal conversion materials. This paper reviews the applications of MXene, black phosphorus (BP), polydopamine/graphene oxide (PDA/GO), and metal-based nanomaterials in bone repair. We also analyze photothermal-based immune regulation, sequential repair strategies, and tumor theranostics. Finally, we discuss current challenges and future trends to guide the design of next-generation smart bone repair materials.

## 1. Introduction

Bone defects caused by trauma, infection, tumor resection, and osteoporosis significantly impact quality of life through complex cellular cascades and microenvironment remodeling [1,2,3]. While autografts and allografts are commonly used, they are limited by donor site morbidity, insufficient supply, and risk of immune rejection. In contrast, conventional bone tissue engineering scaffold plays passive role of supporter, instead of intervention in a damaged microenvironment. To address these limitations, smart stimulus-responsive biomaterials have been developed that can actively modulate the damaged microenvironment in response to external cues. Among these smart biomaterials, there are some NIR light-photosensitive heating materials, which have become known for their very powerful spatial and temporal control options.

PTT converts NIR light into heat and has evolved from tumor ablation into a tool for tissue regeneration [1,2,3,4]. Mild hyperthermia (40–42 °C), when applied with precisely controlled exposure duration (typically 5–15 min), promotes bone healing by inducing heat shock protein (HSP) expression, activating Akt signaling, and driving M2 macrophage polarization, while maintaining BMSC viability above 85%. The therapeutic window is narrow: temperatures of 42–43 °C can trigger early apoptotic pathways, and sustained exposure above 45 °C causes necrotic cell death [1,5]. Therefore, the functional viability of bone cells after photothermal treatment must be rigorously assessed. Zhao et al. [1] demonstrated that 42 °C hyperthermia balances inducible Nitric Oxide Synthase (iNOS) and Arginase-1 (Arg1) to shift macrophages from a pro-inflammatory M1 state to a pro-healing M2 phenotype, creating an ideal immune window for bone repair. These mild photothermal effects activate endogenous repair mechanisms at cellular and molecular levels. Building efficient bone repair platforms requires the rational design of photothermal agents based on their classification, physical properties, and conversion mechanisms.

## 2. Method

### 2.1. Literature Search Strategy

This review ensures methodological transparency and reproducibility in literature selection. Comprehensive searches were conducted across PubMed, Web of Science Core Collection, Scopus, and Google Scholar databases using a carefully designed combination of controlled vocabulary (MeSH terms where available) and free-text keywords with Boolean operators. The primary search string was: (“photothermal therapy” OR “PTT” OR “near-infrared photothermal” OR “NIR”) AND (“bone repair” OR “bone regeneration” OR “bone defect” OR “osteogenesis” OR “osseointegration”). Secondary search terms targeting specific materials included: (“MXene” OR “Ti_3_C_2_T_x_” OR “black phosphorus” OR “BP” OR “polydopamine” OR “PDA” OR “graphene oxide” OR “GO” OR “metal-organic framework” OR “MOF” OR “manganese” OR “magnesium” OR “titanium”) AND (“osteogenic differentiation” OR “osteoblast” OR “mesenchymal stem cell” OR “BMSC” OR “bone mineralization” OR “alkaline phosphatase”). The reference lists of all identified review articles were manually screened to capture additional relevant studies not retrieved by the electronic database search. The initial search yielded 847 records, which were subsequently deduplicated using EndNote X9, resulting in 512 unique articles. Two independent reviewers (X.Y. and C.Z.) then performed a rigorous title/abstract screening process.

### 2.2. Study Selection and Eligibility Criteria

**Figure 1:**
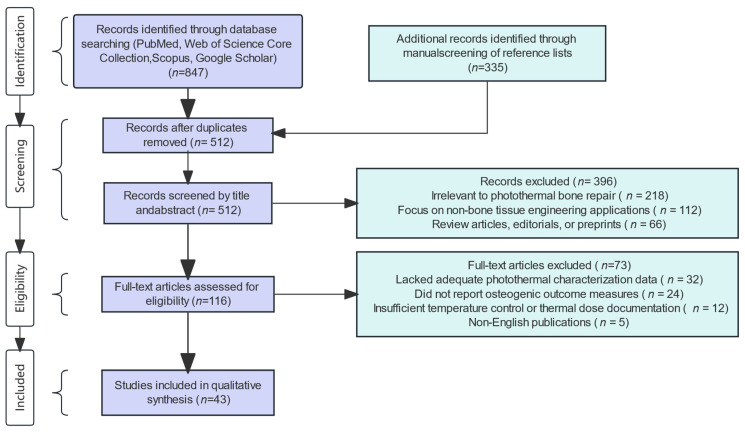
The flow diagram summarizes the literature search and study selection process. From an initial pool of 847 records identified through database searching, 512 unique records remained after deduplication. Following title/abstract screening (396 excluded) and full-text eligibility assessment (73 excluded), 43 studies met all inclusion criteria and were included in this review.

Strict inclusion criteria were applied during the screening phase: studies must (i) employ photothermal materials for bone tissue engineering or bone defect repair applications; (ii) provide quantitative photothermal performance metrics including photothermal conversion efficiency and temperature profiles under NIR irradiation; (iii) report biological outcomes related to osteogenesis, including cell viability, alkaline phosphatase (ALP) activity, mineralization assays, osteogenic gene/protein expression, or in vivo bone formation; and (iv) include detailed methodological descriptions with adequate control groups. This screening phase excluded 396 articles primarily due to irrelevance to photothermal bone repair (*n* = 218), focus on non-bone tissue engineering applications (*n* = 112), or being review articles, editorials, or preprints (*n* = 66). The remaining 116 articles underwent full-text evaluation against more stringent criteria, where an additional 73 studies were excluded for specific reasons: 32 lacked adequate photothermal characterization data, 24 did not report osteogenic outcome measures, 12 provided insufficient temperature control or thermal dose documentation, and 5 were non-English publications. The final 43 included studies were selected based on their scientific rigor, with particular emphasis on those demonstrating methodological innovation, mechanistic insights into photothermal-biological interactions, or clinical relevance for bone defect treatment. While these studies covered four primary material categories (MXenes, black phosphorus, polydopamine/graphene oxide, and metal-based nanomaterials), this review focused on comparing photothermal mechanisms, osteogenic efficacy, and immune-modulatory effects across material types, supplemented by manual reference list screening to provide broader context. The study selection process is summarized in Figure 1.

## 3. Overview of Photothermal Materials

Photothermal materials absorb and convert light into heat with high efficiency under tissue-penetrating NIR (650–1350 nm) light. Integrated into hydrogels, scaffolds, or coatings, these materials act as nano-heaters that produce mild local heat to activate bone repair signaling pathways. Reported photothermal materials for bone repair are categorized into the following groups.

However, a critical limitation of NIR-based photothermal therapy is the restricted light penetration depth through biological tissues. Although the NIR window (650–1350 nm) offers improved tissue penetration compared to visible light, the effective penetration depth through dense cortical bone is typically limited to approximately 2–5 mm, while penetration through thick human soft tissue (e.g., muscle and adipose layers) rarely exceeds 1–2 cm at clinically safe power densities. This poses significant challenges for treating deep-seated bone defects in large animals or human patients, where the defect site may be located several centimeters beneath the skin surface. Strategies to overcome this limitation include the use of NIR-II window (1000–1350 nm) for deeper tissue penetration, fiber-optic light delivery systems for direct irradiation of the defect site, and implantable wireless photothermal devices that eliminate the need for external light sources.

Two-Dimensional Nanomaterials: MXenes (e.g., Ti_3_C_2_T_x_, Nb_2_C) and black phosphorus (BP) are leading examples in this category. MXenes provide high photothermal efficiency, conductivity, and hydrophilicity for integration into 3D-printed scaffolds and conductive hydrogels [5,6]. BP features a tunable bandgap and degrades into phosphate ions that directly aid bone mineralization, making it ideal for sequential repair and theranostics [6,7].

Carbon-based and Biomimetic Materials: GO and PDA offer versatile functionalization. GO uses its planar structure and functional groups to load drugs and growth factors while acting as a photothermal agent and reinforcing phase. PDA forms adhesive coatings on diverse surfaces through self-polymerization and provides melanin-like photothermal conversion with high biocompatibility [8,9].

Metal-based Nanomaterials: This group includes manganese compounds, titanium alloys, magnesium particles, and metal-organic frameworks (MOFs). These materials provide photothermal effects and release bioactive ions like Zn^2+^, Ca^2+^, and Mg^2+^ to stimulate osteogenesis and angiogenesis [10,11]. Some manganese-based materials also exhibit enzyme-like activity to inhibit heat shock proteins and overcome tumor heat resistance at mild temperatures [11].

Other Emerging Agents: Organic dyes like indocyanine green (ICG) serve as photothermal switches to trigger drug release. Additionally, various nanohybrids use synergistic effects between different components to enhance overall performance.

The efficacy of photothermal materials in bone repair stems from the synergy between thermal effects and intrinsic material properties [9,12]. Mild hyperthermia (40–42 °C) upregulates HSPs (HSP70, HSP90) and activates osteogenic signaling pathways, such as PI3K/Akt and MAPK, to drive mesenchymal stem cell differentiation into osteoblasts. This thermal stimulus also shifts macrophage phenotypes from pro-inflammatory M1 to pro-healing M2, optimizing the local immune microenvironment [1,9]. For infected bone defects, high-temperature photothermal effects (>50 °C) disrupt bacterial membranes and biofilms to ensure sterility, while the 45–50 °C range enables selective sterilization that spares mammalian cells. Furthermore, physicochemical traits, including surface functionalization, ion release, and conductivity, independently promote osteogenesis, creating a multi-modal “photothermal-material” therapeutic platform.

Building on these mechanisms, various photothermal systems have been developed to achieve precise bone regeneration (Table 1). This review highlights representative materials and their recent advancements, demonstrating how their diverse compositions and efficient light-to-heat conversion provide versatile strategies for controllable bone repair.

## 4. Applications of Photothermal Materials (MXenes, Black Phosphorus, and Others) in Bone Repair

### 4.1. Two-Dimensional MXene Materials

MXenes are a class of two-dimensional (2D) transition metal carbides, nitrides, and carbonitrides with the general formula M_(n+1)_X_n_T_x_ (where M is an early transition metal, X is carbon and/or nitrogen, and T_x_ represents surface terminations such as -OH, -O, or -F). Their combination of metallic conductivity, hydrophilic surface chemistry, and strong near-infrared absorption makes them uniquely suited as photothermal agents for biomedical applications.

MXenes, particularly Ti_3_C_2_T_x_, have emerged as premier photothermal platforms for bone repair due to their exceptional light-to-heat conversion efficiency, high conductivity, and surface functional groups. Current research highlights their integration into various scaffolds, hydrogels, and coatings to enable multiple therapeutic functions.

Three-dimensionally printed porous scaffolds represent the primary architecture for MXene integration. Huang et al. functionalized ceramic scaffolds with Ti_3_C_2_T_x_ to spatiotemporally modulate the transition from early-stage inflammation to late-stage mineralization via NIR timing [14]. Similarly, Han et al. combined DOPA-SDF1 with poly(ε-caprolactone)/MXene (PCL/MXene) scaffolds to drive synergistic chemotactic and photothermal regeneration [13]. To reduce antibiotic dependence, Tan et al. integrated the natural compound berberine with MXenes, achieving light-activated antibacterial activity and sustained bone growth through a “natural product-nanomaterial” synergy [17].

In hydrogels and coatings, MXenes provide unique multi-physical stimuli. Zhang et al. developed Poly (L-lactic acid)/MXene (PLLA/MXene) piezoelectric hydrogels that utilize mechano-electro-thermal coupling to promote jawbone regeneration via mitophagy-dependent immunometabolic reprogramming [6]. For alveolar bone healing, injectable MXene/Ag-HA hydrogels have demonstrated superior regenerative capacity [16]. In addition, surface coatings incorporating PDA-coated MXene nanosheets have been shown to drive osteogenesis while polarizing macrophages toward an anti-inflammatory M2 phenotype, establishing an immunological basis for photothermal bone repair [18].

Advanced structural designs further extend the utility of these materials. Super-assembled niobium-based (Nb) MXene frameworks exhibit osseointegration efficiency outperforming traditional titanium [19], while ultra-strong MXene films offer a unique combination of mechanical resilience and regenerative signaling [15]. As highlighted by Zorrón et al., the integration of 2D nanomaterials into hydrogel systems offers a robust “treatment-regeneration” paradigm [5]. Taken together, MXenes serve as more than antibacterial agents; they act as physical triggers for stem cell differentiation and immune modulation. Their unique nanotopography and degradation kinetics provide long-term support for tissue ingrowth, positioning them as a foundation for next-generation multifunctional bone repair materials.

### 4.2. Black Phosphorus-Based Photothermal Materials

BP is a 2D nanomaterial for bone engineering, distinguished by its layer-dependent bandgap, high photothermal efficiency, and inherent biodegradability. Critically, its degradation into non-toxic phosphate ions provides an endogenous source for biomineralization [7]. Recent research has diversified BP’s applications across hydrogels, scaffolds, and multifunctional coatings.

In hydrogel systems, BP enables precise microenvironmental modulation. Zhang et al. developed BP-based photothermal hydrogels that utilize mild hyperthermia to alleviate oxidative stress and restore bone homeostasis in osteoporotic conditions [29]. Beyond simple heating, BP can act as a light-triggered switch for drug delivery; for instance, BP-Sr microspheres allow for on-demand, power-dependent release of Sr^2+^ ions to achieve personalized temporal dosing [25]. Additionally, electrosprayed BP@alginate microspheres have been designed to mimic bone matrix vesicles, significantly accelerating mineralization and regenerative kinetics [24].

Scaffolds and coatings further leverage BP’s photothermal and structural properties. BP-doped silk fibroin nanofibers function as localized “thermal platforms” to drive osteogenic differentiation [21], while multilayered EGCG-BP/polyimide scaffolds support vascularized repair in large-scale infected defects, using Epigallocatechin gallate (EGCG) to stabilize BP against premature degradation [22]. In the realm of implantology, BP-doped silk coatings for artificial ligaments [28] and electrophoretically deposited BP/hydroxyapatite/chitosan coatings for titanium implants [27] have demonstrated superior graft-to-bone healing and osseointegration.

The application of BP is particularly transformative in integrated cancer theranostics. BP quantum dot nanocomposites have been developed to simultaneously ablate residual bone tumors and initiate osteogenesis [20]. Similarly, fructose-mineralized BP and mussel-inspired PDA-BP coatings on Poly (phthalazinone ether nitrile ketone) (PPENK) implants offer a triple-function platform, combining antitumor therapy, anti-infection properties, and bone regeneration [23,26]. These “mussel-inspired” strategies exploit the synergy between polydopamine’s adhesion and BP’s photothermal capacity, providing a reliable approach for post-surgical bone tumor management.

In summary, BP’s natural compatibility with bone mineral chemistry and its efficient light-to-heat conversion make it a cornerstone of multifunctional bone repair. However, enhancing its long-term stability in complex physiological environments remains a pivotal challenge for future clinical translation.

### 4.3. Polydopamine and Graphene Oxide-Based Materials

PDA and GO serve as versatile building blocks for photothermal bone-repair platforms. PDA, characterized by its oxidative self-polymerization and melanin-like structure, exhibits universal adhesion to virtually any substrate, while its abundant catechol moieties facilitate secondary functionalization. GO complements these attributes with an expansive specific surface area and a robust photothermal response, making it an ideal candidate for drug loading and mechanical reinforcement.

In multifunctional coatings and scaffolds, PDA is frequently employed as both a stabilizing “bio-glue” and a photothermal sensitizer. For instance, Xia et al. utilized PDA to anchor MXene nanosheets, significantly enhancing the composite’s photothermal efficiency and biocompatibility [18]. This mussel-inspired chemistry has also been applied to cell-membrane-mimetic coatings for anti-infection [31] and bioactive microsphere scaffolds that coordinate the delivery of multiple growth factors [30]. By integrating carbon-based synergy, Chen et al. developed GO/black phosphorus-functionalized collagen scaffolds; under NIR irradiation, this system triggers in situ biomineralization and activates the PI3K/Akt signaling pathway to effectively resolve infected bone defects [32]. The synergy between PDA and black phosphorus has also been validated for its triple-modal efficacy in antitumor, antibacterial, and regenerative therapies [23].

Beyond structural roles, these materials enable stimuli-responsive signaling. Cheng et al. engineered a photothermal-responsive system where NIR light serves as a “molecular switch” to trigger the precise release of nitric oxide (NO), thereby coupling osteogenesis with angiogenesis [12]. This strategy highlights a shift toward gas-signaling-mediated, drug-independent repair.

Typically, these materials function as high-performance additives or coatings rather than standalone bulk scaffolds. When integrated with traditional frameworks such as collagen or PCL, they provide a flexible means to endow implants with light-controlled bioactivity. Collectively, the superior surface chemistry and photothermal conversion of PDA and GO provide the technical foundation for the next generation of intelligent, multifunctional, and precisely regulated bone-regenerative materials.

### 4.4. Metal-Based and Other Photothermal Materials

Beyond the traditional focus on 2D materials, emerging inorganic architectures, including manganese phosphates, specialized titanium alloys, magnesium-based ceramics, and MOFs, have introduced strategies for photothermal bone repair. These materials transition from passive scaffolds to active, multi-functional systems that integrate ion-based signaling, catalytic activity, and structural mechanics with light-responsive therapy.

In the field of implant surface engineering, Li et al. developed a Zn/Ca-doped manganese phosphate coating on titanium substrates [10]. This design establishes a “photothermal-ion” crosstalk, where NIR-induced heat provides rapid antibacterial action while the sustained release of Zn^2+^ and Ca^2+^ ions biochemically orchestrates osteogenesis. Similarly, Cai et al. leveraged the intrinsic photothermal response of Ti-Mo-Zr alloys, demonstrating that the combination of surface nanotopography and active ion release can autonomously drive both disinfection and bone induction [33].

Innovations in bone tumor management have moved toward overcoming the physiological limitations of conventional thermal therapy. Xu et al. utilized 3D-printed magnesium peroxide (MgO_2_) scaffolds to couple photothermal ablation with sustained oxygenation, mitigating hypoxia to enhance the regenerative microenvironment post-tumor resection [34]. Most of note, Zhao et al. reported an Mn_3_O_4_-reinforced hydrogel that exploits nanozymatic activity to disrupt cellular metabolism [11]. By consuming Adenosine Triphosphate (ATP) and inhibiting HSPs, this platform achieves effective tumor ablation at mild temperatures (40–43 °C), thereby protecting surrounding healthy tissue and facilitating immediate osteogenesis. This “catalytic-photothermal” synergy represents a significant departure from high-heat strategies, offering a safer clinical pathway for post-surgical recovery.

In addition, MOFs and programmable structures are advancing the precision of bone repair. Byun et al. demonstrated that MOF-based composite hydrogels can seamlessly transition from tumor ablation to tissue regeneration [35]. Taking this a step further, Yin et al. engineered an NIR-regulated, programmable periosteal scaffold capable of sequential therapeutic delivery [36]. By following a strictly timed “antibacterial → osteogenesis → remodeling” logic, this system ensures that each phase of the healing cascade is optimally supported.

Despite the promising advances described above, several critical gaps and limitations in the current research landscape must be acknowledged. First, there is a notable lack of standardized characterization protocols for photothermal performance, making direct comparisons between studies difficult. Parameters such as laser power density, irradiation time, and distance from light source vary considerably across published reports. Second, most studies rely on small animal models (rats and rabbits) with relatively thin overlying soft tissue, which does not adequately represent the light attenuation challenges encountered in large animals or human patients. Third, the long-term fate and potential systemic toxicity of photothermal nanomaterials remain insufficiently investigated, particularly for non-biodegradable agents such as MXenes and GO. Fourth, the interaction between photothermal effects and the mechanical loading environment of load-bearing bones has rarely been examined. Finally, the economic feasibility and scalability of manufacturing complex photothermal scaffolds for clinical deployment have not been addressed.

## 5. Shared Biological Mechanisms Underlying Photothermal Effects

Biological mechanisms in the context of photothermal bone repair refer to the integrated set of cellular and molecular responses, including thermally induced signaling cascades, immune cell reprogramming, and extracellular matrix remodeling, that collectively translate photothermal stimuli into regenerative outcomes.

Despite the structural and compositional diversity of these materials, their pro-osteogenic mechanisms converge upon several fundamental biological principles. Deciphering these shared pathways is essential not only to elucidate why photothermal intervention is effective but also to guide the design of superior next-generation platforms. To this end, the core biological mechanisms of photothermal bone repair can be categorized into three synergistic dimensions:

1. Mild Hyperthermia and Immunomodulatory Priming

Thermal stimulation serves as the primary catalyst for establishing a pro-regenerative microenvironment. Data from Zhao et al. [1] indicate that mild hyperthermia (~42 °C) dynamically modulates the iNOS/Arg1 balance, driving macrophage polarization from a pro-inflammatory M1 phenotype toward an anti-inflammatory and pro-osteogenic M2 phenotype [1]. This “healing window” is molecularly initiated by the upregulation of HSPs. Specifically, HSP70 and HSP90 stabilize and activate Akt, triggering the PI3K/Akt signaling cascade, which ultimately drives the expression of master osteogenic transcription factors such as Runx2 and Osterix. Furthermore, photothermal effects can engage neuro-immune pathways, modulating local catecholamine levels and neuropeptide release to indirectly refine the bone microenvironment [3]. This immune activation is intrinsically linked to angiogenesis: growth factors (e.g., VEGF and PDGF) secreted by M2 macrophages stimulate neovascularization, which provides the necessary metabolic conduits and progenitor cells for bone formation [37].

2. Pathogen Clearance and Niche Decontamination

In cases of infected or high-risk defects, NIR-induced localized heating ensures a sterile regenerative niche by disrupting bacterial membrane integrity and eradicating recalcitrant biofilms [38,39,40]. Innovative “cloaking” strategies now allow for the targeted thermal ablation of specific pathogens, preserving the commensal flora and reducing collateral tissue damage [4]. Beyond physical disruption, recent findings suggest that photothermal stress can induce ferroptosis-like programmed cell death in bacteria, a non-canonical mechanism that effectively bypasses conventional antibiotic resistance [41].

3. Multi-modal Synergy with Inherent Material Properties

The efficacy of photothermal therapy is amplified when coupled with the material’s intrinsic bioactive properties, such as the release of therapeutic ions (Ca^2+^, Mg^2+^, Zn^2+^, Sr^2+^) or piezoelectric/conductive stimulation. A prime example is the integration of gas signaling, where light-triggered NO release activates the sGC-cGMP pathway, coupling vasodilation with enhanced osteogenesis [12].

## 6. Emerging Interdisciplinary Directions: From Tissue Repair to Sequential Intelligent Theranostics

Sequential Intelligent Theranostics refers to an integrated therapeutic strategy in which a single material platform performs multiple functions, such as diagnosis, tumor ablation, antibacterial action, and bone regeneration, in a temporally programmed sequence. By modulating external stimuli (e.g., NIR light power density, irradiation duration, and wavelength), each therapeutic function can be activated at the optimal time point to match the biological requirements of distinct healing phases, thereby achieving on-demand, phase-specific treatment within one unified system.

### 6.1. Bone Tumor Theranostics Integration

Photothermal materials enable programmable, sequential treatments for bone tumor repair by adjusting light parameters on a single platform. Yin et al. designed NIR-regulated scaffolds that release antimicrobial peptides and growth factors to match the specific timing of inflammation and remodeling during bone healing [36]. Huang et al. used MXene-modified ceramic scaffolds for spatiotemporal control where early mild heat suppresses inflammation and later signals trigger bone cell differentiation [14]. Byun et al. combined photothermal effects with chemical therapy to prevent tumor recurrence and support vascularized bone growth at the same time [35].

Current design rules for next-generation scaffolds focus on integrating therapeutic and regenerative functions as a core principle [40]. Bigham et al. used black phosphorus to transition from tumor removal to bone activation as the material degrades under light [20]. Li et al. used PDA-BP coatings to add antitumor and antibacterial properties to implants for direct bone repair [23]. Zhong et al. showed that photothermal immunotherapy can clear lung cancer bone metastasis and repair bone simultaneously [42]. These advancements move biomaterials from passive supports toward smart “theragenerative” systems [5]. Programmable photothermal materials integrate oncology with bone repair to provide efficient solutions for complex bone tumor management.

### 6.2. Intelligent Sequential Regulation Strategies

Bone repair is a programmed process consisting of inflammation involving hematoma formation, repair characterized by angiogenesis and callus growth, and remodeling which converts woven bone to lamellar bone [18,19]. Each stage requires unique microenvironmental signals ranging from immune activation for tissue clearance to vascular growth for cell migration and enzyme regulation for structural optimization. Precise control over photothermal penetration, duration, and power density enables phase-specific treatment protocols. Adjusting NIR parameters on a single platform satisfies the sequential requirements of each healing stage to achieve on-demand therapy.

**Figure 2:**
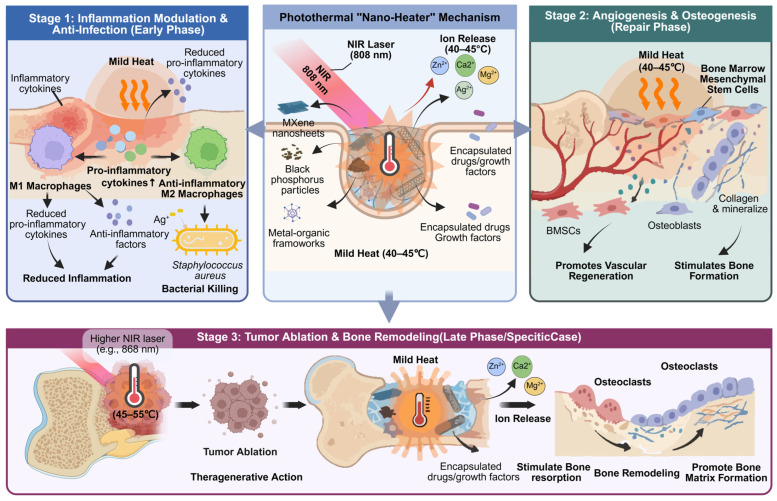
The three phases of bone repair-inflammatory phase, repair phase, and remodeling phase. The inflammatory phase (Stage 1) is characterized by hematoma formation and immune cell recruitment, during which mild photothermal stimulation (40–42 °C) promotes M2 macrophage polarization. The repair phase (Stage 2) involves angiogenesis and osteogenic differentiation via PI3K/Akt and MAPK pathways. The remodeling phase (Stage 3) converts woven bone into lamellar bone, supported by sustained ionic release from degrading photothermal materials. Importantly, tumor ablation is not a sequential step within a single treatment protocol. In clinical practice, high-temperature ablation (>50 °C) is performed as a separate therapeutic stage prior to bone repair, followed by a recovery period before initiating mild photothermal regeneration (40–42 °C). The figure layout illustrates the full spectrum of photothermal applications across all phases of bone healing rather than prescribing a rigid treatment sequence. Dashed arrows indicate potential therapeutic interventions at distinct clinical timepoints. Created in BioRender. Yan, X. (2026) https://BioRender.com/fulo54n.

Regarding the placement of tumor ablation in the remodeling phase (Stage 3) of Figure 2, this reflects a clinical scenario in which post-operative residual tumor cells are managed after initial bone repair has commenced, rather than representing a sequential step within a single protocol. In practice, photothermal tumor ablation and bone regeneration are typically performed as separate therapeutic stages, where high-temperature ablation (>50 °C) is applied first to eradicate tumor cells, followed by a recovery period before initiating mild photothermal bone repair (40–42 °C) to minimize damage to regenerating tissue. The dashed arrows in Figure 2 are intended to convey this flexibility, indicating that ablation can occur at clinically appropriate timepoints rather than at a fixed position in the healing cascade.

Yin et al. designed a programmable periosteal scaffold to demonstrate sequential therapy through a three-stage light plan that releases antimicrobial peptides and growth factors and maintains enzyme activity to match the healing phases of inflammation, repair, and remodeling [36]. Huang et al. used MXene-modified ceramic scaffolds to inhibit NF-κB pathways early to reduce inflammation and activate PI3K/Akt pathways later to promote mineralization [14]. Wang et al. developed NIR-II hydrogels that penetrate deep into tissue to treat irregular bone defects [43]. These advancements move biomaterials from passive supports to active participants that deliver biophysical and biochemical signals on a set timeline. Next-generation materials must use photothermal control to coordinate treatment and regeneration timing for better outcomes [5,6,9]. Intelligent regulation gives photothermal materials the ability to manage complex bone repair through personalized and efficient regeneration paths.

While intelligent sequential regulation represents a major conceptual advance, several obstacles hinder its practical implementation. The primary challenge lies in achieving real-time, in vivo temperature monitoring with sufficient spatial and temporal resolution to enable closed-loop feedback control. Current approaches rely predominantly on ex vivo thermal imaging or implanted thermocouples, neither of which provides the comprehensive temperature mapping needed for precise dose control in three-dimensional defect sites. Additionally, the biological heterogeneity among patients, including differences in bone mineral density, vascular supply, immune status, and comorbidities such as diabetes or osteoporosis, means that a single programmed sequence may not be universally optimal. The integration of multiple stimuli-responsive functions within a single material system increases fabrication complexity and introduces potential failure modes. Addressing these challenges through interdisciplinary collaboration among materials scientists, bioengineers, and clinicians will be critical.

## 7. Conclusions and Outlook

Photothermal materials reshape bone repair strategies through their structural tunability and functional integration. MXenes and black phosphorus are widely studied as photothermal agents because of their strong near-infrared absorption and unique degradation properties. Polydopamine and graphene oxide provide surface modification and loading capabilities while metal-based materials and metal-organic frameworks offer mechanical support and ionic bioactivity. Packaging these units into injectable hydrogels or 3D-printed scaffolds creates a necessary path for clinical translation.

Several technical and biological challenges remain. Precise temperature control in deep bone defects is difficult and requires closed-loop systems with internal feedback to avoid overheating. Unstable materials like black phosphorus need better antioxidant strategies to maintain their effectiveness over time. Most research uses small animal models and lacks standardized light parameters or evidence from large animal critical-sized defects. Optimal temperature windows and light protocols change depending on the defect type and require more systematic studies on dose-response relationships. The long-term impact of photothermal effects on the immune microenvironment and chronic inflammation risks are also not fully understood.

Future bone repair will move toward intelligent and personalized systems that integrate real-time temperature monitoring and adaptive drug delivery. Smart implants will combine artificial intelligence to predict light parameters and multimodal imaging to track healing progress. These integrated platforms shift photothermal bone repair toward precision medicine to provide solutions for complex bone defects.

## Figures and Tables

**Table 1:** Classification, characteristics, and applications of major photothermal materials for bone repair.

Material Category	Representative Materials	Photothermal Conversion Mechanism	Core Advantages	Typical Application Forms
2D MXene Materials	Ti_3_C_2_T_x_, Nb_2_C	Strong NIR absorption and efficient photothermal conversion by 2D nanosheets	Ultrahigh photothermal conversion efficiency; excellent conductivity; abundant surface functional groups for easy functionalization	3D-printed ceramic/polymer scaffolds, piezoelectric hydrogels, injectable hydrogels, polymer coatings [5,6,13,14,15,16,17,18,19]
Black Phosphorus Based	BP nanosheets (BPNSs), BP quantum dots (BPQDs)	Layer-number-tunable direct bandgap gives NIR absorption	Biodegradable (degradation product phosphate ions participate in bone mineralization); strong photothermal conversion capability	Photothermal-responsive hydrogels, self-assembled microspheres, electrospun fiber coatings, injectable bone cement [6,7,20,21,22,23,24,25,26,27,28,29]
Polydopamine Materials	PDA coatings, PDA nanoparticles	Inherent NIR absorption and photothermal conversion of melanin-like structure	Adheres to almost any surface (universal glue); excellent biocompatibility; easy secondary functionalization	Implant surface coatings, composite scaffold modification layers, mussel-inspired microspheres [8,9,18,23,30,31]
Graphene Oxide Materials	GO, rGO	NIR absorption by sp^2^ conjugated regions in 2D carbon skeleton	Huge specific surface area; rich oxygen-containing functional groups; can load drugs/growth factors	Composite collagen scaffolds, nanocomposite hydrogels [8,32]
Manganese-based Materials	Mn_3_O_4_, manganese phosphate (MnPO_4_)	d-d electron transition and NIR absorption	Possess oxidase/peroxidase-mimetic catalytic activity; release osteogenesis-related ions (Zn^2+^, Ca^2+^)	Bifunctional hydrogels (tumor ablation + osteogenesis), titanium implant coatings [10,11]
Titanium/Magnesium Alloy	Ti-Mo-Zr alloy, magnesium-based ceramics (MgO_2_)	Surface plasmon resonance effect of metallic nanostructures	Inherent mechanical support; release bioactive ions (Mg^2+^); can integrate oxygen-releasing function	3D printed porous scaffolds, nanostructured alloy surface platforms [33,34]
Metal-Organic Frameworks	ZIF-8 composite nanohybrids	Synergistic NIR absorption by organic ligands and metal nodes	Large specific surface area; tunable pore size; easy drug loading	Multifunctional composite hydrogels, programmable periosteal scaffolds [35,36]
Emerging Composite Agents	GO/BP composites, PDA-BPs integrated coatings	Synergistically enhanced NIR absorption by multiple components	Complementary advantages (e.g., GO enhances BP stability; PDA toughens BP and imparts adhesion)	Functionalized collagen scaffolds, biomimetic coatings [22,23,32]
Organic Small Molecule	ICG	NIR absorption by π-π conjugated structure of organic dyes	Clinically approved; clear metabolic pathway	Embedded in thermosensitive systems as photothermal switch to trigger drug release [8,12]

## Data Availability

No new data were created or analyzed during this study. Data sharing is not applicable.

## Abbreviations

The following abbreviations are used in this manuscript:

NIR	Near-Infrared
PTT	Photothermal Therapy
BP	Black phosphorous
PDA	Polydopamine
GO	Graphene Oxide
HSP	Heat Shock Protein
iNOS	inducible Nitric Oxide Synthase
Arg1	Arginase-1
MOFs	Metal–organic frameworks
ICG	Indocyanine green
BPNSs	BP nanosheets
BPQDs	BP quantum dots
PCL	poly(ε-caprolactone)
PLLA	Poly (L-lactic acid)
Nb	Niobium-based
EGCG	Epigallocatechin gallate
PPENK	Poly (phthalazinone ether nitrile ketone)
NO	Nitric oxide
ATP	Adenosine Triphosphate
